# Advancements in facial implantology: a review of hydroxyapatite applications and outcomes

**DOI:** 10.3389/fsurg.2024.1409733

**Published:** 2024-07-18

**Authors:** Martin Kauke-Navarro, Leonard Knoedler, Samuel Knoedler, Ali Farid Safi

**Affiliations:** ^1^ Department of Surgery, Division of Plastic and Reconstructive Surgery, Yale New Haven Hospital, Yale School of Medicine, New Haven, CT, United States; ^2^ Craniologicum, Center for Craniomaxillofacial Surgery, Bern, Switzerland; ^3^ Department of Oral and Maxillofacial Surgery, Charité – Universitätsmedizin Berlin, Corporate Member of Freie Universität Berlin, Humboldt-Universität zu Berlin, and Berlin Institute of Health, Berlin, Germany; ^4^ Medical Faculty, University of Bern, Bern, Switzerland

**Keywords:** face, apatite, hydroxyapatite, augmentation, reconstruction

## Abstract

**Background:**

The search for an ideal bone substitute in reconstructive surgery has led to the exploration of various materials, with hydroxyapatite (HaP) emerging as a promising candidate due to its biocompatibility, osteoconductive properties, and structural similarity to human bone. Despite its potential, there is a paucity of data on the long-term safety and efficacy of HaP in facial skeletal reconstruction and augmentation.

**Methods:**

We conducted a systematic review following PRISMA 2020 guidelines, searching PubMed/MEDLINE, Google Scholar, CENTRAL, and Web of Science databases for studies on hydroxyapatite facial implants in reconstruction and augmentation.

**Results:**

Our search yielded 12 studies that met our inclusion criteria, encompassing 74 patients treated with HaP implants for various indications including facial fractures/defects, aesthetic facial balancing, and after tumor resection. The studies reported on outcomes such as implant integration, complications, aesthetic results, and patient satisfaction, with a general trend indicating positive outcomes for the use of HaP in facial reconstruction.

**Conclusion:**

Hydroxyapatite appears to be a viable and effective material for facial skeletal reconstruction and augmentation, offering benefits in terms of biocompatibility, osteoconductivity, and patient outcomes. However, limitations such as low mechanical strength and the need for further research on long-term safety and efficacy were identified. This review underscores the potential of HaP in craniofacial surgery while highlighting areas for future investigation.

## Introduction

Autologous options for craniofacial reconstruction such as bone or cartilage grafts present the standard of care for selected patients and pathologies. However, autologous reconstruction has limitations such as donor site morbidity and limitations in terms of availability, unpredictable resorption rates, increased cost and operating time, as well as limitations in terms of contouring (1, 2). Alloplastic materials such as polymers (e.g., Poly-ether-ether-ketone), metals (e.g., Titanium) and ceramics (e.g., Hydroxyapatite) have been used with varying success (2, 3).

Bioceramics are advanced inorganic non-metallic solid ceramic materials that include a large number of different materials (e.g., hydroxyapatite, alumina, zirconia, bioactive glasses) and are typically inorganic, have a crystalline structure, are biocompatible but brittle (low crack resistance), low or no toxicity and allow integration into the recipient (4). Bioceramics are thus often used in the field of bone tissue engineering (5). Hydroxylapatite or Hydroxyapatite (HaP) has, due its similarity to the naturally occurring calcium phosphate apatite component of bone [main inorganic component of human bone (approximately 70 mass %), bonds with collagen fibers (30 mass %)], become an important asset in the field of bone tissue engineering and hard tissue reconstruction (4, 6–8).

HaP is available by processing naturally occurring biologic products (e.g., human/bovine bone) or synthetically produced HaP by various methods (available as granules or blocks or even injectable material) (7, 9). One key advantage of HaP over other alloplastic implant materials is the nontoxic nature, high biocompatibility due to similarity with native bone, low immunogenicity, an inherent osteoconductive capability and role as a scaffold for bony ingrowth which allows transformation of the material through a sequence of events into ECM and ultimately bone (7, 10). One of the main issues that was raised is the brittleness and low mechanical strength of HaP (11). However, other implant materials such as PEEK, Titanium, Silicone, Porous Polyethylene will persist *in situ* and are not exchanged for bone (2, 12).

For the use in cranioplasty, HaP is a well-studied material with relatively low complication rates (sometimes reported similar to autologous bone and similar to better risk profile than MMA, PEEK and Titanium)) however limitations due to low mechanical strength (13, 14). To date, there is little evidence on the safety and efficacy of hydroxyapatite facial implants. The herein presented systematic review aims to fill this knowledge gap by summarizing available evidence on the use of HaP for facial skeletal reconstruction and augmentation.

## Methods

**Figure 1:**
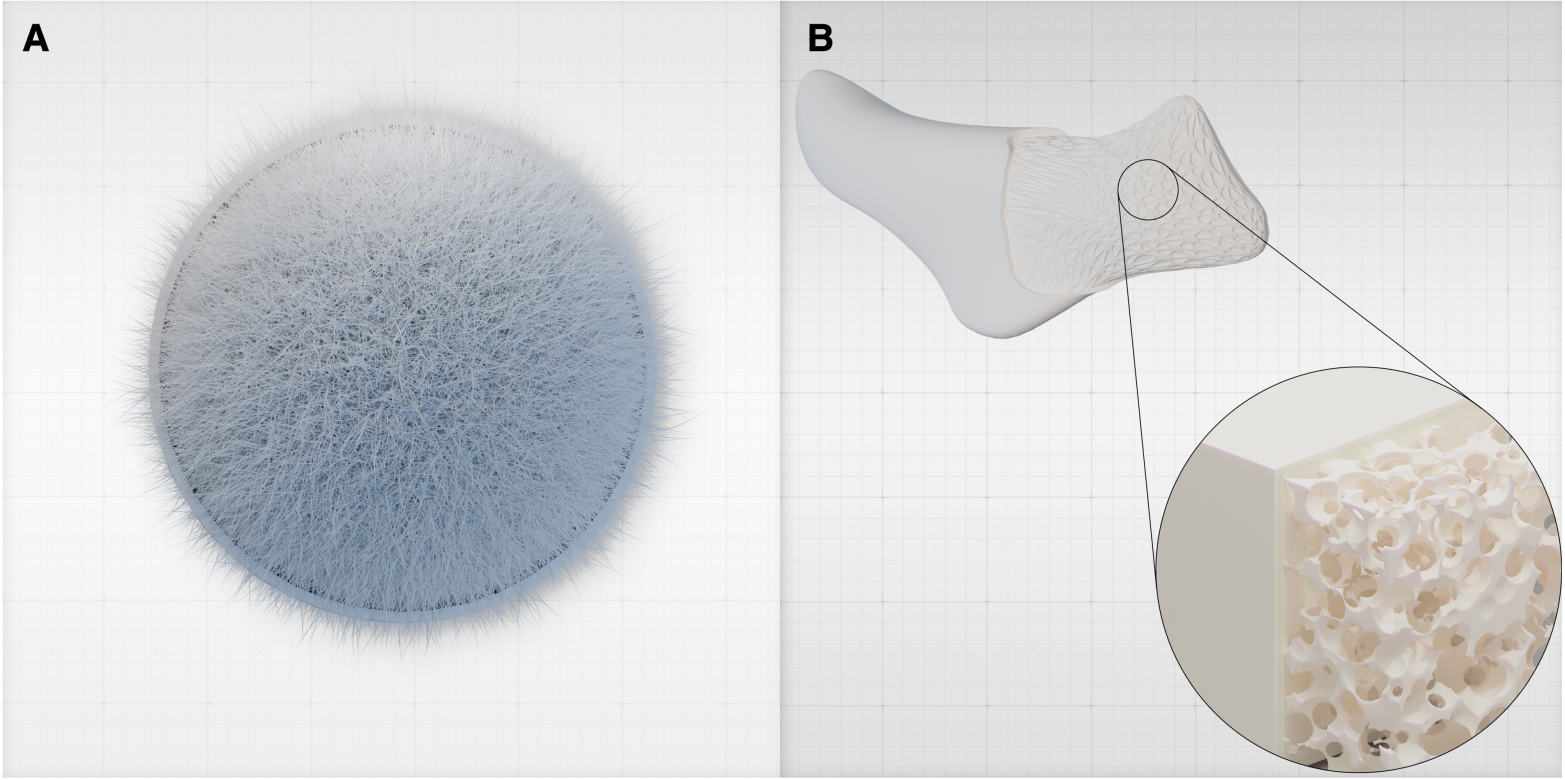
(**A**) Hydroxyapatite is a crystalline inorganic material that constitutes the primary mineral component of natural bone, providing strength and rigidity. It is bound to organic components such as collagen and other proteins. (**B**) HaP can be tailored into custom-designed, patient-specific implants (PSIs) based on pre-operative imaging to precisely address the patient's anatomical needs. Using additive manufacturing and bioprinting techniques, the implant's porosity and architecture are meticulously engineered to enhance its characteristics. For example, a macro-porous architecture and a gyroid lattice structure (15) are utilized. These custom-made HaP implants can potentially replace other alloplastic implants, obviating the need for foreign materials such as PEEK or silicone.

The methodology of performing a systematic review was previously described by our work group (12). In brief, we systematically reviewed the literature using PubMed/MEDLINE, GoogleScholar, CENTRAL, and Web of Science from database inception to March 15th 2023 for studies investigating the use of hydroxyapatite facial implants (Figure 1). The following search terms were used: (Hydroxylapatite OR Hydroxyapatite) AND implant AND (reconstruction OR augmentation) AND (face OR facial OR zygoma OR malar OR mandible OR maxilla OR chin OR gonial OR frontal OR calvarium OR cranium OR cranioplasty).

**Figure 2:**
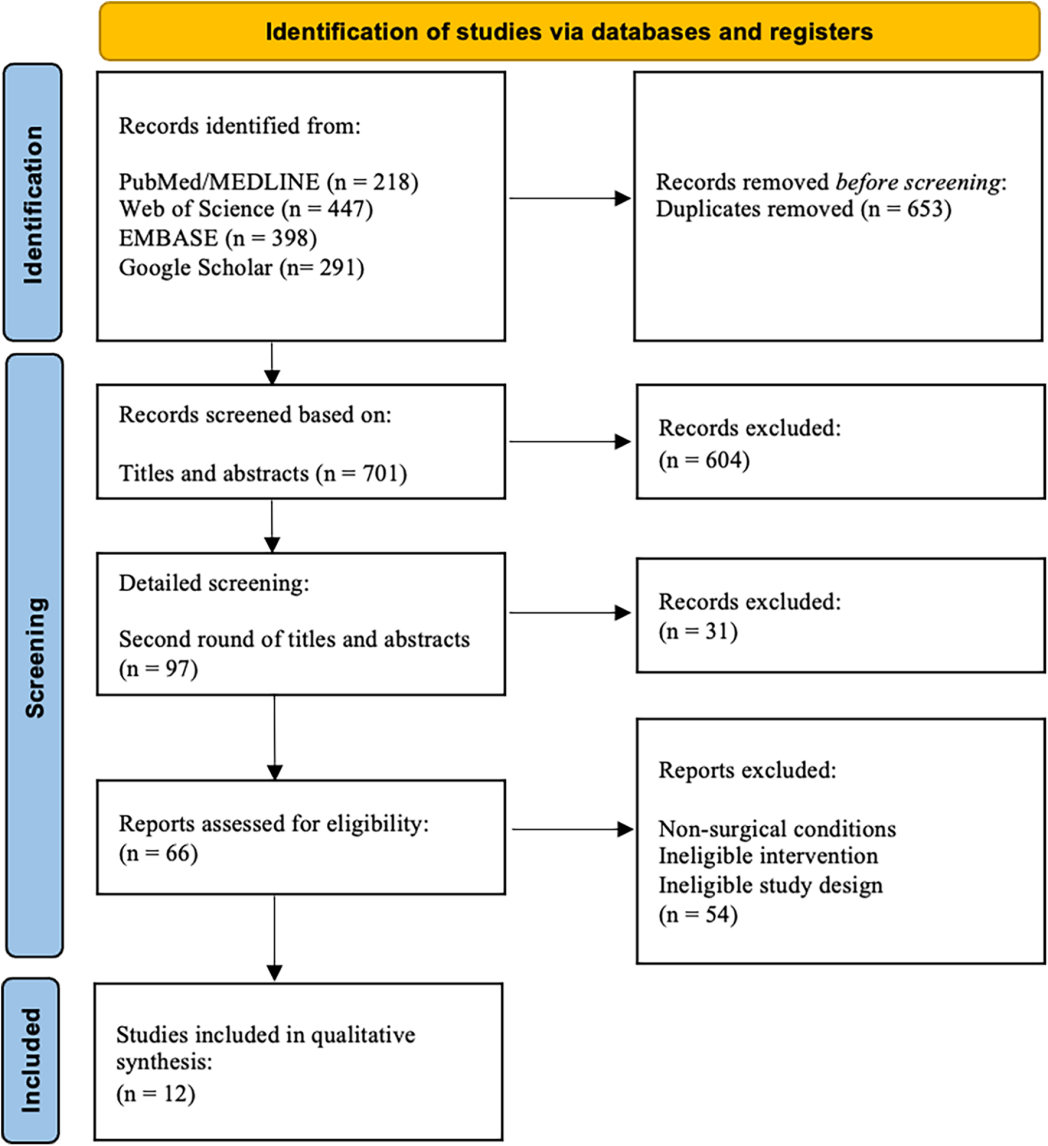
PRISM flow chart.

We excluded all non-human studies or articles in a language other than English. Studies investigating outcomes related to HaP cranioplasties, maxillary sinus augmentations, soft tissue filler, and dental implants were excluded. The herein presented study was conducted in accordance with the Preferred Reporting Items for Systematic Reviews and Meta-Analyses (PRISMA) 2020 guidelines (16) (Figure 2). The study presents a descriptive review of the data gathered by the abovementioned search strategy. A meta-analysis was not performed due to the heterogeneity of the outcome parameters.

Two reviewers (M.K.-N. and L.K.) independently screened the titles and abstracts of the articles using Covidence (17). A subsequent full-text review was performed manually for abstracts that had been considered eligible. Any disagreements were discussed with a third reviewer (A.F.S.) and resolved by consensus.

## Results

### General study characteristics

The comprehensive literature search retrieved 1,354 articles, of which 12 met our inclusion and exclusion criteria (18–29) (Table 1). The level of evidence was IV in 9 studies (75%). In summary, a total of 74 patients were included. All studies were published between 1997 and 2024. The included patients' ages covered a broad range, ranging from as young as 9 years old to as old as 65 years old patients. The first study was published in 1997 and the latest in 2024.

**Table 1:** Overview of research findings on hydroxyapatite implants and descriptive analysis.

Year	Author	Study design	Number of patients (age range, y)	Material	Follow up months (mean, range)	Reconstructed area	Complications	Postoperative implant fracture requiring RTOR	Reason for surgery	Functional outcome	Aesthetic outcome	Implant measurements	PSI
2024	Systermans et al.	Cohort study	13 (20–52)	HaP [CAD/CAM, CERHUM SA(Liege, Belgium)]	9 (1–22)	Mandible (symphysis, body, angle), Zygoma, Orbital floor	Fractured implant (4/13), implant exposure requiring removal (1/13)	No	Genioplasty (5), reconstructive (4), aesthetic augmentation (4)	Increased osseointegration and bone formation, reduction of cephalgia and cervicalgia	Subjectively satisfied	–	Yes
2016	Kattimani et al.	Case series	4 (20–65)	Hydroxyapatite block (Biograft, IFGL Ceramics, Kolkata, India)	One patient 60 months, unknown for the other 3	Mandible	None reported	No	Mandibular defects	New bone formation and bone-implant-bridging	–	–	Yes
2012	He et al.	Comparative study	64 of which only 19 received HAP as part of their reconstruction (NA)	5 HaP, 2 Titanium mesh with HaP, 12 Titanium mesh “either Ha/Medpor”	1	Orbital fractures	Not specified for patients who received HaP. For the whole cohort: temporal atrophy in 4, ectropion *n* = 3, infection *n* = 1	No	Restoration of enopthalmic defects	Technique dependent successful enopthalmos correction and zygomatic reduction	Perfect symmetry in 41 patients	–	Yes
2012	Tieghi et al.	Case report	1 (50)	40% Hydroxyapatite + 60% hemihydrate calcium sulfate – cerament (Bonesupport AB, Lund, Sweden)	48	Fracture involving NOE, frontal bone and supraorbital rim	None reported	No	Contouring of boney forehead irregularities	Successful defect coverage	Subjectively satisfied	–	No
2012	Wehrli et al.	Case report	1 (9)	Hydroxylapatite ceramic implant (Custom Bone, Finceramica, Faenza (RA), Italy	16	Frontal region	None reported	No	Juvenile psammomatoid ossifying fibroma	Successful defect coverage	Subjectively satisfied	–	Yes
2011	Chai et al.	Case series	12 (18–45)	Epoxied maleic acrylate/hydroxyapatite compound (CAD/CAM, china)	1–24	Fronto-orbital nasal defects	None reported	No	Traumatic fronto-orbital nasal defects	Successful defect reconstruction	Subjectively satisfied	–	Yes
2011	Li et al.	Case report	1 (27)	Nanoscale hydroxyapatite/polyamide composite (*n* = HA/PA) CAD/CAM	24	Mandible	None reported		Mandibular defect	Improved maximal mouth opening reach and decreased mouth-opening deviation	Subjectively satisfied	–	Yes
2001	Ducic et al.	Comparative study	14 (NA)	Titanium mesh intraoperatively filled with hydroxyapatite cement (Stryker-Leibinger, Kalamazoo, MI)	6 months on average	Orbit	In 3 patients (Dacryocystitis (2), ptosis (1)).		Orbit reconstruction	–	–	–	No
1999	Pessa et al.	Case report	1 (52)	Fast set porous hydroxyapatite	NA	Pyriform aperture	None reported		Augmentation of the pyriform aperture	–	Rejuvenated facial features	2.5 g per side	No
1999	Lemke et al.	Case series	5 (24–33)	Porous HA block (Interpore, Irvine, CA)	57 (46–65)	Orbital floor	None reported, mild residual enopthalmus/vertical globe dystopia in 3 patients		Orbital floor fracture	Mild residual enophthalmos persisted in three patients	–	10 mm	No
1999	Eguchi et al.	Case report	1 (18)	Hydroxyapatite, not further specified	36	Orbital rim	None reported		Soft and hard tissue atrophy – coup de sabre scleroderma	Good bone fixation and no evidence of postoperative HAP absorption	Subjectively satisfied	–	No
1997	Hirano et al.	Case series	2 (42–46)	Multiporous hydroxyapatite block (Asahi Optical Company, Tokyo)	8–48	Zygoma	None reported		Intraosseous hemangiomas	Successful defect reconstruction	Subjectively satisfied	20 × 30 × 7 mm	No

### Clinical indications

In 10 studies (83%), HAP implants were used to repair facial fractures/defects. One study (8%) reported on HAP implants for improving facial aesthetics and one study (8%) reported a combination of fracture repair and improvement of facial aesthetics (21, 26). The most common clinical indications for surgical reconstruction were neoplastic conditions (*n* = 4, 33%), followed by bone fractures (*n* = 4, 33%) (17–20, 24, 28, 30, 31).

### Facial regions

Four studies (33%) investigated HAP implants for surgical reconstruction of the orbital region including the orbital wall, orbital floor, and supraorbital rim (18, 28, 30, 32). Two studies (17%) focused on the mandibular region (17, 21). One publication (8%) studied the frontal region (31). Further, there were five studies (42%) that included multiple facial regions (19, 20, 24, 26, 33).

### Implant composition and measurements

Overall, only three (25%) studies provided a more detailed description of the exact composition of the HAP implant (20, 21, 24). Tieghi et al. described the implantation of cerament consisting of 60% calcium sulfate and 40% HAP (24). Li et al. reported the use of a HAP-polyamide implant without providing any additional information on the material composition (21). Further, He et al. described HAP implants combined with a titanium mesh (18). Yet, the authors did not include any additional data on the implant material. Similar to the biomaterial composition, implant measurements were only included in three (25%) studies (19, 26, 28). Implant diameter ranged from 1 to 35 mm, while the implant weight was only reported in one study (8%) (19, 26, 28). Production of hydroxyapatite based patient specific implants via CAD/CAM processing was specifically described in 3 studies (18, 23, 24).

### Intra- and postoperative complications

The post-op follow-up period ranged from 1 to 65 months (18, 28). The majority of articles did not report any complications (8/12). Intraoperative fractures of HaP PSIs were reported by Systermans et al. in 4 out of 13 patients. In all these cases the HaP PSIs were still implanted and did not require exchange for another implant. None of the 12 articles report on postoperative implant fractures for which the patient required return to the OR. Postoperative implant exposure requiring removal was noted in 1 patient (18). Ducic et al. described the use of titanium mesh filled with HaP intraoperatively and noted dacryocystitis in 2 patients and ptosis in 1 patient after orbital defect repair as a complication. In one study, porous HaP blocks were used for orbital floor reconstruction with 3 out of 5 patients presenting with postoperative residual enopthalmus and/or vertical globe dystopia. One study (He et al. 2012) reported on the combined outcome of HaP and other implant materials without specifically assessing HaP related complications.

### Functional outcomes

The most common outcome measurement tool was CT scans. There were no standardized parameters of outcome measurement. CT scans were used in eleven studies (92%) to assess bone-implant interaction (e.g., bony ingrowth, CT density of the HAP implant) (17–21, 24, 28, 30–33). He et al. assessed functional outcomes based on enophthalmos correction and zygomatic reduction in comparison to scans taken prior to implant placement. Successful enophthalmos correction was defined as postoperative globe projection, whilst zygomatic reduction was described as “good” alignment of the zygomaticofrontal and sphenoid sutures, inferior orbital rim, and zygomatic arch (20). Li et al. determined the implant functionality based on the maximal pre- and postoperative mouth-opening distance after repair of a mandibular defect (24).

### Aesthetic outcome and patient satisfaction

In total, *n* = 9 studies (75%) included data on aesthetic results, while *n* = 2 (17%) studies reported on patient satisfaction (18–21, 24, 26, 31–33). Pessa et al. reported a more youthful facial appearance as indicated by a higher alar base position in the Frankfurt horizontal, increase in the nasolabial angle and superior rotation of the nasal tip (26). Systermans et al. proposed improved palpebral fold aesthetics (i.e., less sunken eyes, enhanced support of the ocular prosthesis) as an indicator of good aesthetic results, without providing any further definition of “improved palpebral fold aesthetics” (18). Moreover, He et al. measured the globe projection and zygomatic reduction via CT and revealed a good projection in 83% of cases using combined materials vs. 20% of cases using HAP-only implants. “Good projection” was defined as ≤2 mm, mild enophthalmos as ≤3 mm, and moderate enophthalmos as ≤4 mm (20). No further information on the measurement of patient satisfaction was provided in the other articles (17, 28, 30).

## Discussion

Hydroxyapatite has emerged as a relevant material used in craniofacial reconstruction and balancing of the craniofacial skeleton (2). Aside from bony augmentation (e.g., maxillary sinus augmentation prior to dental implant placement) and reconstruction, HaP has also been used as a soft tissue injectable filler (e.g., Radiesse) (34, 35). Despite the widespread use of HaP for balancing of facial features, little is known about the safety and efficacy as a material used for reconstruction and aesthetic implant based augmentation of the face. Recently, Systermans et al. published their case series on HaP PSIs used in reconstruction of the zygoma, mandible, orbital floor and augmentation of the mandibular angle which supports the safety of HaP PSIs in facial reconstructive surgery (18). However, there is a lack of long-term studies and comprehensive reviews assessing the safety and overall durability of HaP facial implant. To fill this gap, we systematically reviewed the literature.

Hydroxyapatite demonstrates some clear biologic advantages over other alloplastic materials (4). The high biocompatibility has been demonstrated in various animal studies utilizing different compositions (e.g., solid, granules) and porosities of HaP (4). HaP is considered to be a bioactive and bioresorbable ceramic, and forms a strong chemical bond at the interface with normal bone (4). Dense HaP formations are essentially stable *in situ*; however, porosity is associated with resorption. Mechanical stress was shown to be a factor that may influence regeneration of bone at the defect site with osteoclast-driven resorption of HaP followed by osteoblastic regeneration to re-establish bone (4). In one included study by Ducic et al., 4 patients underwent a biopsy after HaP implantation for repair of orbital defects. The specimens showed osseous ingrowth into the hydroxyapatite cement at all time points between 6 months to 3 years postoperatively (25). Hydroxyapatite ocular implants offer some interesting insights into the bio-acceptance of the material. Porous HaP ocular implants appear to promote fibrovascular tissue ingrowth even when implanted into the orbit without significant signs of foreign body reactions (32). This inherent biocompatibility and osteoconductive feature with the ability to osseointegrate distinguishes HaP from other alloplastic materials (18).

Another potential advantage of HaP can be seen in the pediatric population. To date, despite high complication rates (up to 32% in cranioplasty) autologous bone grafts remain standard of care for craniofacial reconstruction (36). HaP has been used as an alternative or in revision cases due to its similarity with natural bone and, compared to other alloplastic materials (e.g., PEEK), its unique ability to expand with growth (37, 38).

In the herein presented systematic review of the literature we identified 74 patients who received HaP based facial implants between 1997 and 2024. The primary use of HAP implants was for repair of facial fractures/defects, with a significant portion of studies also addressing neoplastic conditions, bone fractures, and congenital facial defects. Only 1 study used HaP PSIs in 6 patients for aesthetic balancing of facial features (18). Most studies used HaP for the orbital complex (orbital wall, floor) and less studies presented the use of HaP for load bearing areas of the mandible/maxilla. This coincides with the known disadvantage of HaP and low mechanical resilience.

Overall, the small number of patients over a period of 27 years underlines that HaP implants and specifically patient individualized implants have not been extensively studied in the literature. This may point towards an inherently difficult handling of the implants itself as suggested by Systerman et al. who reported on frequent implant fractures of the defect specific implants. There's a notable gap in reporting outcomes for HaP facial implants, with most studies offering short follow-up periods and lacking long-term data. The inherent fragility of HaP implants is a major concern, highlighting the necessity for extended observation to understand their long-term integration. Despite their fragility and intraoperative fracture risk, postoperative fractures with significant clinical consequences seem rare once the implants are correctly placed. Further research is essential to elucidate the durability of HaP implants over time. Their suitability appears to be dependent on the implantation site, being potentially effective for non-load-bearing areas like malar or mandibular angle augmentation, but less so for repairing load-bearing sections.

The relatively low revision surgery rate, despite the incidence of complications, may indicate that many postoperative issues can be managed conservatively or do not significantly impact the overall outcome. However, this could also point to a potential underreporting of the need for revision surgeries or to limitations in follow-up duration, which varies significantly among the studies. In general, there was no standardized outcome reporting system, nor were some patients followed sufficiently to adequately report on long term outcomes.

Another important observation is the observed wide range of HaP compositions and manufacturing protocols. Very few studies utilized patient specific implants and dedicated CAD/CAM manufacturing protocols. In fact, only one study (Systermans et al.) describe in detail their PSI manufacturing protocol.

The integration of CAD/CAM technology has revolutionized the precision with which implants and prostheses can be designed, allowing for tailor-made solutions that fit the unique contours of individual patients' anatomy with unprecedented accuracy. Customization of facial implants is vital for achieving not only functional but also aesthetic outcomes, ensuring that reconstructions are not only medically successful but also visually appealing and harmonious with the patient's features (39). Moreover, the manufacturing advancements have streamlined the production process, making it faster, more cost-effective, and capable of producing more complex shapes and structures that were previously unachievable. Concurrently, bioprinting technology's emergence plays a crucial role, especially in hydroxyapatite applications. It allows for the creation of complex, organic structures, significantly impacting scaffold development (7, 11, 15). This synergy between bioprinting and hydroxyapatite technology promises to refine regeneration techniques, offering more natural, effective reconstruction solutions with enhanced aesthetic outcomes.

## Conclusion

The comprehensive review of literature on hydroxyapatite (HAP) implants for facial reconstruction reveals their predominant use in repairing facial fractures and defects, with a notable focus on the orbital and maxillary regions. Despite the challenges in standardizing outcome measurements and managing postoperative complications, HAP implants show promising functional and aesthetic outcomes, emphasizing the need for further research on biomaterial composition and objective outcome measures. Furthermore, the review unveils a lack of long term follow up studies after HaP based facial implant reconstruction, highlighting the necessity for studies with extended durations to accurately assess outcomes. Additionally, there is a lack of comparative studies that evaluate the safety, efficacy of HaP based facial reconstruction and augmentation to other materials and the current gold standard (autologous grafting). Larger more homogenous and controlled trials are necessary to better define the safety profile of HaP implants for facial reconstruction and aesthetic augmentation to allow more thorough analysis of outcomes.

## References

[1] Yaremchuk MJ. Atlas of Facial Implants. London: Elsevier Health Sciences (2019).

[2] Kauke-Navarro M Safi A-F. Balancing beauty and science: a review of facial implant materials in craniofacial surgery. Front Surg. (2024) 11:1348140. 10.3389/fsurg.2024.1348140 38327548 PMC10847330

[3] Kauke-Navarro M Knoedler L Deniz C Knoedler S Safi A-F. Early outcomes and risk factors for complications after facial alloplastic implant surgery: an ACS-NSQIP study. J Plast Reconstr Aesthet Surg. (2024) 90:209–14. 10.1016/j.bjps.2024.02.021 38387417

[4] Dubok VA. Bioceramics — yesterday, today, tomorrow. Powder Metall Met Ceram. (2000) 39:381–94. 10.1023/A:1026617607548

[5] Okada M Furuzono T. Hydroxylapatite nanoparticles: fabrication methods and medical applications. Sci Technol Adv Mater. (2012) 13:064103. 10.1088/1468-6996/13/6/064103 27877527 PMC5099760

[6] Narasaraju TSB Phebe DE. Some physico-chemical aspects of hydroxylapatite. J Mater Sci. (1996) 31:1–21. 10.1007/BF00355120

[7] Kattimani VS Kondaka S Lingamaneni KP. Hydroxyapatite—past, present, and future in bone regeneration. Bone Tissue Regen Insights. (2016) 7:BTRI.S36138. 10.4137/BTRI.S36138

[8] Gibson IR. Natural and synthetic hydroxyapatites. In Biomaterials Science. Elsevier (2020). p. 307–17. 10.1016/B978-0-12-816137-1.00023-4

[9] Mohd Pu’ad NAS Abdul Haq RH Mohd Noh H Abdullah HZ Idris MI Lee TC. Synthesis method of hydroxyapatite: a review. Mater Today Proc. (2020) 29:233–9. 10.1016/j.matpr.2020.05.536

[10] Fiume E Magnaterra G Rahdar A Verné E Baino F. Hydroxyapatite for biomedical applications: a short overview. Ceramics. (2021) 4:542–63. 10.3390/ceramics4040039

[11] Bal Z Kaito T Korkusuz F Yoshikawa H. Bone regeneration with hydroxyapatite-based biomaterials. Emergent Mater. (2020) 3:521–44.

[12] Kauke-Navarro M Knoedler L Knoedler S Deniz C Safi A-F. Surface modification of PEEK implants for craniofacial reconstruction and aesthetic augmentation—fiction or reality? Front Surg. (2024) 11:1351749. 10.3389/fsurg.2024.1351749 38481611 PMC10936457

[13] Khalid SI Thomson KB Maasarani S Wiegmann AL Smith J Adogwa O Materials used in cranial reconstruction: a systematic review and meta-analysis. World Neurosurg. (2022) 164:e945–63. 10.1016/j.wneu.2022.05.073 35623608

[14] Liu L Lu S-T Liu A-H Hou W-B Cao W-R Zhou C Comparison of complications in cranioplasty with various materials: a systematic review and meta-analysis. Br J Neurosurg. (2020) 34:388–96. 10.1080/02688697.2020.1742291 32233810

[15] Van Hede D Liang B Anania S Barzegari M Verlée B Nolens G 3D-printed synthetic hydroxyapatite scaffold with in silico optimized macrostructure enhances bone formation *in vivo*. Adv Funct Materials. (2022) 32:2105002. 10.1002/adfm.202105002

[16] Page MJ McKenzie JE Bossuyt PM Boutron I Hoffmann TC Mulrow CD The PRISMA 2020 statement: an updated guideline for reporting systematic reviews. Syst Rev. (2021) 10:89. 10.1186/s13643-021-01626-4 33781348 PMC8008539

[17] Kellermeyer L Harnke B Knight S. Covidence and rayyan. J Med Libr Assoc. (2018) 106. 10.5195/jmla.2018.513

[18] Systermans S Cobraiville E Camby S Meyer C Louvrier A Lie SA An innovative 3D hydroxyapatite patient-specific implant for maxillofacial bone reconstruction: a case series of 13 patients. J Craniomaxillofac Surg. (2024) 52:S1010518224000738. 10.1016/j.jcms.2024.02.026 38461138

[19] Kattimani VS Chakravarthi PS Prasad LK. Biograft block hydroxyapatite: a ray of hope in the reconstruction of maxillofacial defects. J Craniofac Surg. (2016) 27:247–52. 10.1097/SCS.0000000000002252 26745193

[20] He D Li Z Shi W Sun Y Zhu H Lin M Orbitozygomatic fractures with enophthalmos: analysis of 64 cases treated late. J Oral Maxillofac Surg. (2012) 70:562–76. 10.1016/j.joms.2011.02.041 21752509

[21] Tieghi R Consorti G Clauser LC. Contouring of the forehead irregularities (washboard effect) with bone biomaterial. J Craniofac Surg. (2012) 23:932–3. 10.1097/SCS.0b013e3182505593 22627408

[22] Wehrli L Zweifel N Weil R Altermatt S. Juvenile psammomatoid ossifying fibroma of the forehead, radical resection, and defect coverage with a hydroxyl-apatite composite—a case report. Eur J Pediatr Surg. (2012) 22:479–84. 10.1055/s-0032-1313349 22648192

[23] Chai G Zhang Y Ma X Zhu M Yu Z Mu X. Reconstruction of fronto-orbital and nasal defects with compound epoxied maleic acrylate/hydroxyapatite implant prefabricated with a computer design program. Ann Plast Surg. (2011) 67:493–7. 10.1097/SAP.0b013e318201fddf 21629112

[24] Li J Hsu Y Luo E Khadka A Hu J. Computer-aided design and manufacturing and rapid prototyped nanoscale hydroxyapatite/polyamide (n-HA/PA) construction for condylar defect caused by mandibular angle ostectomy. Aesth Plast Surg. (2011) 35:636–40. 10.1007/s00266-010-9602-y 20972567

[25] Ducic Y. Three-dimensional alloplastic orbital reconstruction in skull base surgery. Laryngoscope. (2001) 111:1306–12. 10.1097/00005537-200107000-00031 11568560

[26] Pessa JE Peterson ML Thompson JW Cohran SC Garza JR. Pyriform augmentation as an ancillary procedure in facial rejuvenation surgery. Plast Reconstr Surg. (1999) 103:683–6. 10.1097/00006534-199902000-00050 9950560

[27] Lemke BN Kikkawa DO. Repair of orbital floor fractures with hydroxyapatite block scaffolding. Ophthal Plast Reconstruct Surg. (1999) 15:161–5. 10.1097/00002341-199905000-00004 10355833

[28] Eguchi T Harii K Sugawara Y. Repair of a large ‘coup de sabre’ with soft-tissue expansion and artificial bone graft. Ann Plast Surg. (1999) 42:207–10. 10029489

[29] Hirano S Shoji K Kojima H Omori K. Use of hydroxyapatite for reconstruction after surgical removal of intraosseous hemangioma in the zygomatic bone. Plast Reconstruct Surg. (1997) 100:86–90. 10.1097/00006534-199707000-00015 9207663

[30] Arvanitis P Stratoudakis A Alexandrou C. Secondary orbital implant insertion in an anophthalmic patient after orbital reconstruction. Orbit. (2007) 26:275–7. 10.1080/01676830601168967 18097967

[31] Boxer Wachler BS Holds JB. Difficulties with hydroxyapatite orbital implants in two patients with dysfunctional levator/superior rectus muscle complex. Ophthal Plast Reconstruct Surg. (1997) 13:252–5. 10.1097/00002341-199712000-00004 9430301

[32] Baumgarten D Wojno T Taylor A. Evaluation of biomatrix hydroxyapatite ocular implants with technetium-99m-MDP. J Nucl Med. (1993) 34:467–8. 8382742

[33] Huang Z-L Ma L. Restoration of enophthalmos in anophthalmic socket by HTR polymer. Ophthal Plast Reconstruct Surg. (2005) 21:318–21. 10.1097/01.iop.0000170410.23157.0e 16052153

[34] Allard RHB Swart JGN. Orbital roof reconstruction with a hydroxyapatite implant. J Oral Maxillofac Surg. (1982) 40:237–9. 10.1016/0278-2391(82)90320-2 6279810

[35] Attenello NH Maas CS. Injectable fillers: review of material and properties. Facial Plast Surg. (2015) 31(01):029–34. 10.1055/s-0035-1544924 25763894

[36] Piitulainen JM Kauko T Aitasalo KMJ Vuorinen V Vallittu PK Posti JP. Outcomes of cranioplasty with synthetic materials and autologous bone grafts. World Neurosurg. (2015) 83:708–14. 10.1016/j.wneu.2015.01.014 25681593

[37] Alkhaibary A Alharbi A Alnefaie N Oqalaa Almubarak A Aloraidi A Khairy S. Cranioplasty: a comprehensive review of the history, materials, surgical aspects, and complications. World Neurosurg. (2020) 139:445–52. 10.1016/j.wneu.2020.04.211 32387405

[38] Spennato P Canella V Aliberti F Russo C Ruggiero C Nataloni A Hydroxyapatite ceramic implants for cranioplasty in children: a retrospective evaluation of clinical outcome and osteointegration. Childs Nerv Syst. (2020) 36:551–8. 10.1007/s00381-019-04423-6 31786632

[39] Kamata M Sakamoto Y Kishi K. Foreign-body reaction to bioabsorbable plate and screw in craniofacial surgery. J Craniofac Surg. (2019) 30:e34–6. 10.1097/SCS.0000000000004945 30475293

